# OA22 Are we in sight of disease control – climbing a mountain of challenge with Systemic Onset Juvenile Idiopathic arthritis

**DOI:** 10.1093/rap/rkae117.022

**Published:** 2024-11-05

**Authors:** Nandita Pai, Samundeeswari Deepak, Kishore Warrier, Kapil Gargh

**Affiliations:** Queens Medical Centre, Nottingham, United Kingdom; Queens Medical Centre, Nottingham, United Kingdom; Queens Medical Centre, Nottingham, United Kingdom; Queens Medical Centre, Nottingham, United Kingdom

## Abstract

**Introduction:**

Juvenile idiopathic arthritis (JIA) can have a different trajectory in different patients and some patients can have difficult to control arthritis that changes its profile over time. Despite the advances in treatment with biologics, there are still patients with severe disease profile needing multiple treatments including combination of bio-DMARDs. There is lack of evidence in the literature regarding combining biologics to treat arthritis. After trial of various treatment options, we have been pushed to the corner with this challenging case and have had to try biologic combinations as a precursor to the next step which may be bone marrow transplant.

**Case description:**

Our patient presented at the age of three years in September 2018 with history of progressively worsening pain, stiffness and swelling of multiple joints for four weeks and recurrent high grade fever, six weeks after an episode of chicken pox. Her examination revealed florid polyarthritis affecting both large and small joints. She did not have any rashes, mucocutaneous bleeds, hepatosplenomegaly or serositis. She was diagnosed with systemic onset juvenile idiopathic arthritis. Bone marrow biopsy at the time revealed no evidence of malignancy.

She was treated with intravenous steroids and Methotrexate. Tocilizumab infusions were added on, fortnightly initially and then stretched to monthly. It took over 2 years to achieve optimum disease control during which time she needed multiple intravenous and intra-articular steroids.

Her disease profile changed after the first two years to a more polyarticular pattern with no fever although she does have raised inflammatory markers during flares. We trialled Infliximab infusions for three months alongside Methotrexate but a lack of response made us switch back to Tocilizumab.

Despite regular treatment, she continued to have multiple flares needing steroids. We swapped Tocilizumab to Abatacept infusions alongside Methotrexate. As this failed to control her flares, she moved on to subcutaneous Anakinra in October 2022 alongside subcutaneous Methotrexate.

Although Anakinra seemed promising for the first five months, our patient started having recurrent flares needing further steroids. She developed a prolonged chest infection with a wet cough in September 2023 and had 2 courses of oral and intravenous antibiotics. During this time, she had two flares of arthritis. A HRCT Chest done showed no inflammatory lung involvement but ongoing consolidation for which she was treated with two weeks of intravenous antibiotics. Her induced sputum culture was negative and repeat HRCT after the antibiotics was clear.

**Discussion:**

Given the fact that she had eight flares in the last year and clearly suboptimal control of arthritis despite various treatment strategies, we discussed her at a Bone marrow transplant (BMT) multidisciplinary team meeting (MDT). The consensus was to trial a combination two biologics before considering BMT. We commenced her on Tofacitinib with Anakinra, and in the last two months we have swapped Anakinra back to Tocilizumab infusions as the monthly flares persisted.

The patient and family were kept informed of the management plans throughout this period. We have checked in with the family at various difficult points as well as liaised with school to ensure that our patient’s involvement in activities and her mental health are taken care of and offered support via nurses, play specialists and therapists.

We believe some of the flares over the year could have been triggered by the prolonged chest infection, but this did not explain all the flares. We have screened her for lingering infections including a dental screen for cavities and urine screen to rule out Urinary tract infections as a probable trigger. We have sent a genetic panel and IL18 levels. The question is should we be escalating to BMT just yet, or is there something else we could do to achieve optimal disease control in this young person.

**Key learning points:**

• Disease profile can change from systemic to polyarticular type in children with Systemic juvenile idiopathic arthritis.

• There is lack of guidance on combining biologic agents to treat children with JIA and criteria for bone marrow transplant. A consensus guidance on this would be beneficial to the paediatric rheumatology community.

• It is important to involve the family and young person in all the treatment decisions, aid them to make an informed choice on treatment plans in challenging situations and support them in a holistic manner in ensuring physical, mental and emotional well being.

